# Supplementary material for: Modeling glioblastoma heterogeneity as a dynamic network of cell states
Source: Mol Syst Biol. 2021 Sep 16;17(9):e10105. doi: 10.15252/msb.202010105 (PMC8444284; doi:10.15252/msb.202010105)
Supplement: Supplementary file 5 — Source Data for Figure 3 [file MSB-17-e10105-s001.zip › Figure3A_sourcedata/GSEA_3065/hallmarks_state6.GseaPreranked.1623416308702/gsea_report_for_na_neg_1623416308702.html]

Report for na\_neg 1623416308702 [GSEA]

| GS  follow link to MSigDB | GS DETAILS | SIZE | ES | NES | NOM p-val | FDR q-val | FWER p-val | RANK AT MAX | LEADING EDGE || 1 | HALLMARK\_EPITHELIAL\_MESENCHYMAL\_TRANSITION | Details ... | 117 | -0.67 | -2.78 | 0.000 | 0.000 | 0.000 | 1080 | tags=48%, list=12%, signal=54% |
| 2 | HALLMARK\_E2F\_TARGETS | Details ... | 185 | -0.47 | -2.07 | 0.000 | 0.000 | 0.000 | 2158 | tags=47%, list=25%, signal=61% |
| 3 | HALLMARK\_ANGIOGENESIS | Details ... | 20 | -0.72 | -2.04 | 0.000 | 0.000 | 0.000 | 718 | tags=50%, list=8%, signal=54% |
| 4 | HALLMARK\_CHOLESTEROL\_HOMEOSTASIS | Details ... | 59 | -0.53 | -1.90 | 0.000 | 0.001 | 0.003 | 1287 | tags=37%, list=15%, signal=43% |
| 5 | HALLMARK\_PROTEIN\_SECRETION | Details ... | 88 | -0.48 | -1.88 | 0.000 | 0.001 | 0.005 | 1212 | tags=30%, list=14%, signal=34% |
| 6 | HALLMARK\_G2M\_CHECKPOINT | Details ... | 179 | -0.42 | -1.82 | 0.000 | 0.003 | 0.017 | 2337 | tags=45%, list=27%, signal=60% |
| 7 | HALLMARK\_COAGULATION | Details ... | 53 | -0.50 | -1.81 | 0.000 | 0.004 | 0.025 | 1050 | tags=28%, list=12%, signal=32% |
| 8 | HALLMARK\_COMPLEMENT | Details ... | 92 | -0.43 | -1.69 | 0.000 | 0.011 | 0.079 | 1651 | tags=28%, list=19%, signal=34% |
| 9 | HALLMARK\_MTORC1\_SIGNALING | Details ... | 171 | -0.39 | -1.69 | 0.000 | 0.010 | 0.080 | 1647 | tags=35%, list=19%, signal=42% |
| 10 | HALLMARK\_APICAL\_SURFACE | Details ... | 20 | -0.59 | -1.67 | 0.015 | 0.010 | 0.086 | 344 | tags=25%, list=4%, signal=26% |
| 11 | HALLMARK\_GLYCOLYSIS | Details ... | 140 | -0.39 | -1.64 | 0.000 | 0.012 | 0.115 | 2210 | tags=37%, list=25%, signal=49% |
| 12 | HALLMARK\_INFLAMMATORY\_RESPONSE | Details ... | 68 | -0.42 | -1.56 | 0.010 | 0.025 | 0.239 | 2187 | tags=46%, list=25%, signal=60% |
| 13 | HALLMARK\_ANDROGEN\_RESPONSE | Details ... | 68 | -0.41 | -1.51 | 0.012 | 0.036 | 0.351 | 2245 | tags=41%, list=26%, signal=55% |
| 14 | HALLMARK\_UNFOLDED\_PROTEIN\_RESPONSE | Details ... | 98 | -0.36 | -1.44 | 0.009 | 0.062 | 0.541 | 1339 | tags=23%, list=15%, signal=27% |
| 15 | HALLMARK\_IL2\_STAT5\_SIGNALING | Details ... | 102 | -0.35 | -1.41 | 0.028 | 0.075 | 0.639 | 1574 | tags=28%, list=18%, signal=34% |
| 16 | HALLMARK\_MITOTIC\_SPINDLE | Details ... | 174 | -0.33 | -1.41 | 0.016 | 0.072 | 0.649 | 751 | tags=17%, list=9%, signal=18% |
| 17 | HALLMARK\_UV\_RESPONSE\_DN | Details ... | 104 | -0.34 | -1.38 | 0.039 | 0.085 | 0.736 | 2213 | tags=38%, list=25%, signal=50% |
| 18 | HALLMARK\_ESTROGEN\_RESPONSE\_LATE | Details ... | 99 | -0.33 | -1.32 | 0.066 | 0.129 | 0.876 | 1155 | tags=21%, list=13%, signal=24% |
| 19 | HALLMARK\_ESTROGEN\_RESPONSE\_EARLY | Details ... | 99 | -0.31 | -1.22 | 0.100 | 0.234 | 0.985 | 1155 | tags=21%, list=13%, signal=24% |
| 20 | HALLMARK\_BILE\_ACID\_METABOLISM | Details ... | 51 | -0.33 | -1.17 | 0.204 | 0.313 | 0.996 | 1820 | tags=33%, list=21%, signal=42% |
| 21 | HALLMARK\_APICAL\_JUNCTION |  | 113 | -0.29 | -1.16 | 0.168 | 0.313 | 0.996 | 1981 | tags=26%, list=23%, signal=33% |
| 22 | HALLMARK\_MYOGENESIS |  | 93 | -0.29 | -1.16 | 0.197 | 0.308 | 0.997 | 1284 | tags=24%, list=15%, signal=27% |
| 23 | HALLMARK\_HEDGEHOG\_SIGNALING |  | 24 | -0.38 | -1.15 | 0.283 | 0.308 | 0.998 | 885 | tags=17%, list=10%, signal=18% |
| 24 | HALLMARK\_IL6\_JAK\_STAT3\_SIGNALING |  | 31 | -0.36 | -1.14 | 0.283 | 0.319 | 0.999 | 2086 | tags=42%, list=24%, signal=55% |
| 25 | HALLMARK\_HYPOXIA |  | 123 | -0.27 | -1.11 | 0.252 | 0.349 | 1.000 | 730 | tags=17%, list=8%, signal=18% |
| 26 | HALLMARK\_KRAS\_SIGNALING\_UP |  | 82 | -0.28 | -1.09 | 0.298 | 0.365 | 1.000 | 1583 | tags=27%, list=18%, signal=32% |
| 27 | HALLMARK\_INTERFERON\_ALPHA\_RESPONSE |  | 55 | -0.30 | -1.09 | 0.293 | 0.353 | 1.000 | 2105 | tags=29%, list=24%, signal=38% |
| 28 | HALLMARK\_XENOBIOTIC\_METABOLISM |  | 94 | -0.25 | -1.00 | 0.464 | 0.537 | 1.000 | 1100 | tags=18%, list=13%, signal=20% |
| 29 | HALLMARK\_INTERFERON\_GAMMA\_RESPONSE |  | 94 | -0.24 | -0.96 | 0.527 | 0.619 | 1.000 | 1253 | tags=18%, list=14%, signal=21% |
| 30 | HALLMARK\_SPERMATOGENESIS |  | 55 | -0.25 | -0.93 | 0.560 | 0.671 | 1.000 | 1852 | tags=25%, list=21%, signal=32% |
| 31 | HALLMARK\_MYC\_TARGETS\_V2 |  | 46 | -0.27 | -0.92 | 0.597 | 0.650 | 1.000 | 2288 | tags=33%, list=26%, signal=44% |
| 32 | HALLMARK\_NOTCH\_SIGNALING |  | 22 | -0.27 | -0.79 | 0.772 | 0.899 | 1.000 | 2395 | tags=36%, list=27%, signal=50% |
| 33 | HALLMARK\_FATTY\_ACID\_METABOLISM |  | 103 | -0.18 | -0.72 | 0.968 | 0.948 | 1.000 | 1856 | tags=22%, list=21%, signal=28% |
Table: Gene sets enriched in phenotype **na **[plain text format]****
